# Druggists Renewing Prescriptions

**Published:** 1868-07

**Authors:** 


Druggists Renewing Prescriptions.—The Academy of Medicine, New York, after a long and impartial discussion of the various points at issue, adopted unanimously the following preamble and resolution, and referred to the State Medical Society:

*Whereas*, The attention of this Academy has been called to the repetition of prescriptions, containiug active ingredients, by druggists, without the written order of physicians; and whereas, serious consequences to patients are liable to ensue; therefore,

*Resolved*, That we respectfully request the druggists of this city not to repeat such a prescription without the written order of a physician, he being the only competent judge of the propriety or necessity of such renewal.

